# An *in cellulo*-derived structure of PAK4 in complex with its inhibitor Inka1

**DOI:** 10.1038/ncomms9681

**Published:** 2015-11-26

**Authors:** Yohendran Baskaran, Khay C. Ang, Praju V. Anekal, Wee L. Chan, Jonathan M. Grimes, Ed Manser, Robert C. Robinson

**Affiliations:** 1 Institute of Molecular and Cell Biology, A*STAR (Agency for Science, Technology and Research), Biopolis, Proteos Building, 61 Biopolis Drive, 8-15, Singapore 138673, Singapore; 2 Department of Biochemistry, Yong Loo Lin School of Medicine, National University of Singapore, Singapore 117597, Singapore; 3 Division of Structural Biology, Wellcome Trust Centre for Human Genetics, University of Oxford, Roosevelt Drive, Oxford OX3 7BN, UK; 4 Diamond Light Source Ltd., Diamond House, Harwell Science and Innovation Campus, Didcot, Oxfordshire OX11 0DE, UK; 5 Institute of Medical Biology, A*STAR, Biopolis, Singapore 138648, Singapore; 6 Department of Pharmacology, Yong Loo Lin School of Medicine, National University of Singapore, Singapore 117597, Singapore

## Abstract

PAK4 is a metazoan-specific kinase acting downstream of Cdc42. Here we describe the structure of human PAK4 in complex with Inka1, a potent endogenous kinase inhibitor. Using single mammalian cells containing crystals 50 μm in length, we have determined the *in cellulo* crystal structure at 2.95 Å resolution, which reveals the details of how the PAK4 catalytic domain binds cellular ATP and the Inka1 inhibitor. The crystal lattice consists only of PAK4–PAK4 contacts, which form a hexagonal array with channels of 80 Å in diameter that run the length of the crystal. The crystal accommodates a variety of other proteins when fused to the kinase inhibitor. Inka1–GFP was used to monitor the process crystal formation in living cells. Similar derivatives of Inka1 will allow us to study the effects of PAK4 inhibition in cells and model organisms, to allow better validation of therapeutic agents targeting PAK4.

## Abstract (web summary)

PAK4 is a metazoan-specific kinase, which acts downstream of the cell polarity regulator Cdc42. Here, Baskaran *et al.* determine the structure of PAK4 bound to the endogenous inhibitor Inka1 from crystals that form spontaneously in mammalian cells overexpressing both proteins.

## Graphical abstract

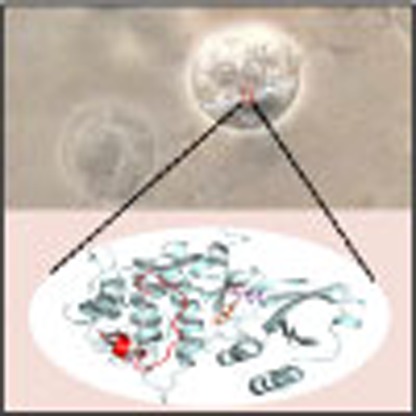

Mammalian PAK isoforms are categorized into two groups on the basis of their structural and biochemical features: the conventional or group I PAKs in human comprise PAKs1–3, whereas the group II PAKs (PAK4–6) are encoded by three genes in mammals^1^,^2^,^3^. PAK4-like kinases are ubiquitously expressed in metazoans but are not found in protozoa or fungi^4^,^5^. This is consistent with PAK4 functioning primarily at cell–cell contacts in mammalian cells^6^, with Cdc42 also being required for adherent junction formation^7^. The phenotype of PAK4-null mice, which is embryonic lethal, involves defects in the fetal heart as well as in neuronal development and axonal outgrowth^8^. The loss of PAK4 prevents proper polarization and thus formation of the endothelial lumen^9^, consistent with defects seen in PAK4−/− mice^10^.

PAK4 is a kinase with strong links to cellular transformation and cancer metastasis, as reviewed^11^. The structural basis for PAK4's preference for serine containing substrate sites has recently been elucidated^12^. We have shown that Cdc42 directly regulates PAK4 activity in mammalian cells through an auto-inhibitory domain (AID) that binds in a manner similar to pseudosubstrates^13^,^14^. This is consistent with the notion that PAK4 lacking residues 10–30 in the Cdc42/Rac interactive binding domain is active^15^. Although PAK1 activation *in vivo* occurs through activation loop Thr-423 phosphorylation^16^, it is notable that PAK4 is constitutively phosphorylated on Ser-474 (ref. 13) and kept in check through the intramolecular association of the AID. The binding of Cdc42 can serve to activate PAK4 in cells, but it is unclear whether there is any autophosphorylation event associated with this activation^13^. As PAK4 does not appear to use adaptors, we investigated the possibility that Inka1, first identified as a PAK4-binding protein in frogs^17^, might fulfill this role.

*In-vivo* protein crystallization is rare with mammalian examples including insulin^18^ and Charcot–Leyden crystals^19^,^20^. The observation that haemoglobin could crystallize on dilution of unpurified red cell lysate^21^ facilitated the advent of protein X-ray crystallography^22^. Only recently have microcrystals generated inside bacterial or insect cells become amenable to X-ray analysis^23^,^24^,^25^. A coral fluorescent protein that forms diffraction-quality micron-sized crystals within mammalian cells^26^ indicates the mammalian cell environment could be a suitable host for a number of proteins, which are not normally crystalline.

Experiments described here suggest that Inka proteins are in fact endogenous inhibitors of PAK4, with the two human Inka isoforms sharing a high degree of sequence identity in the region previously termed the Inca box^17^. Inka1 contains an additional PAK4 inhibitory sequence at its carboxy terminus and either of these sequences can promote crystallization of the catalytic domain of human PAK4 in mammalian cells. An *in-cellulo* protein structure, from X-ray experiments on single crystals formed within a mammalian cell, reveals a hexagonal array of PAK4 subunits that was suggestive of an ability to accommodate other proteins in the lattice. This was demonstrated by fusing Inka1 to green fluorescent protein (GFP). Because of these features the PAK4 array has potential as a protein analogue of ‘crystalline molecular flasks' in which guest molecules can reside to facilitate their X-ray analysis^27^.

## Results

### Inka1 is an endogenous PAK4 inhibitor

**Figure 1:**
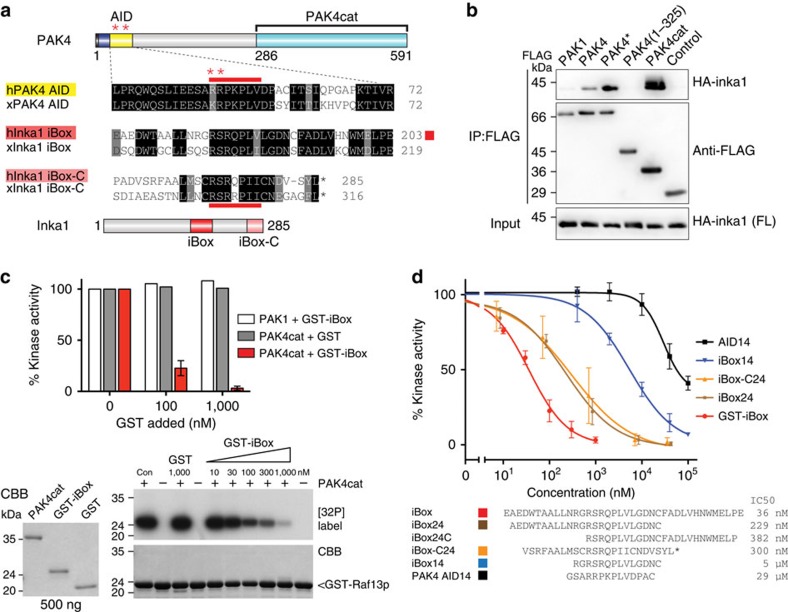
Inka1 is a potent kinase inhibitor. (**a**) PAK4 architecture and alignment of the AID and the Inka1 iBox and iBox-C from frogs and human. Red asterisks indicate activation mutations in PAK4* (RR48/49AE). Red bars indicate pseudosubstrate sequences. (**b**) Co-immunoprecipitation of full-length HA-Inka1 by FLAG-tagged PAK4 constructs. (**c**) Kinase assays using 6His–PAK1 (activated) or PAK4cat, with GST-iBox as indicated. Activity was assessed by the phosphorylation of GST-Raf13 quantified by densitometry (lower right). The quality of the purified proteins is indicated (lower left). (**d**) The inhibition profile of GST-iBox and selected peptides of the iBox and iBox-C (*n*=3, error bars indicate s.e.m.). The IC_50_ values were determined from the intercepts of the graphs.

We previously reported that the Cdc42 effector PAK4 is regulated by an AID (Fig. 1a), which serves to control the constitutively phosphorylated catalytic (PAK4cat) domain^13^. Although Cdc42 upregulates PAK4 activity *in vivo*, this kinase activation cannot be observed using recombinant proteins *in vitro*^14^, indicating other protein(s) might be involved. Indeed, it has been suggested that Src SH3 domain interaction with the core AID sequence might be an alternate means of regulating PAK4^14^, although a cellular Src–PAK4 interaction has not been detected. There are few PAK4-interacting proteins known other than the Cdc42-like GTPases^4^. One *Xenopus* PAK4-binding protein originally identified through a yeast two-hybrid screen is a 30-kDa neural crest enriched protein termed Inka1 (previously Inca^28^,^29^), although the role of this putative adaptor was not determined. The protein is also designated FAM212a and FAM212b in the protein database based on their common central 38 amino acid sequence (166–203) here termed the Inka box (iBox, Fig. 1a).

We decided to investigate the role of human Inka1 by further testing its ability to bind to various PAK4 constructs in mammalian cells. Inka1 bound to an activated PAK4 with a mutated AID (designated PAK4*) significantly better than wild-type PAK4 (Fig. 1b). This suggested that the PAK4 AID limits Inka1 access to the PAK4 catalytic domain (Fig. 1b) with which it interacts^29^. The recombinant 38 amino acid ‘Inka box' (GST-iBox) is a potent of PAK4cat inhibitor *in vitro* (Fig. 1c) but does not affect PAK1, suggesting Inka1 is a specific group II PAK^30^ inhibitor. Inka1 probably acts also on PAK5 and PAK6, as their substrate binding pockets are essentially identical^31^. *In-vitro* measurements indicate GST–Inka1 has a Ki of 30 nM (Fig. 1d), which is comparable with the avidity of protein kinase A (PKA) inhibitor (PKI)^32^. The iBox sequence (Fig. 1a) contains the tripeptide PLV in common with the PAK4-AID, which binds in the substrate-docking site^14^,^33^.

### Inka1 has two functional inhibitory regions

**Figure 2:**
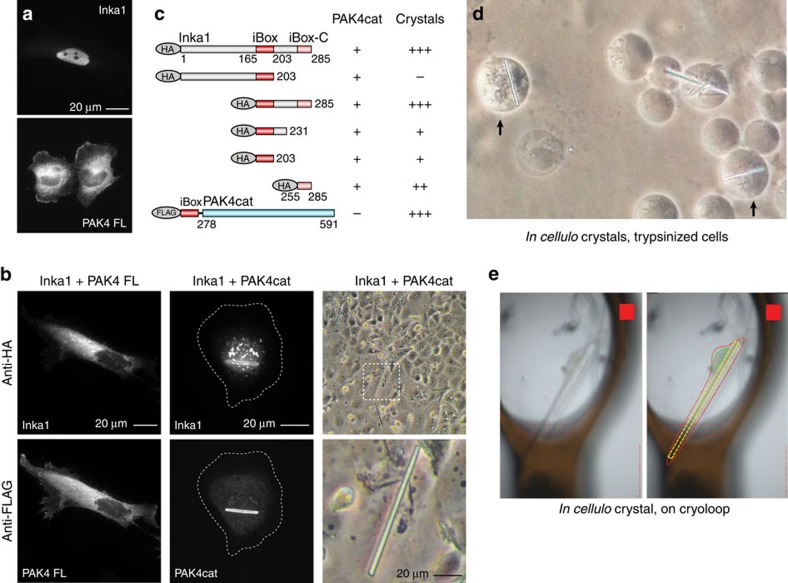
Intracellular PAK4cat:Inka1 crystals. (**a**) Inka1 and PAK4 show nuclear and cytoplasmic localization, respectively. (**b**) Co-expression leads to cytoplasmic enrichment of Inka1 (left panels). Inka1 and PAK4cat co-expression results in intracellular crystals (right panels), which immunostain for both proteins (middle panels). (**c**) Inka1 regions capable of generating co-crystals. A single chain fusion of iBox–PAK4cat efficiently generated intracellular crystals. (**d**) *In-cellulo* crystals of trypsinized cells. (**e**) A single cell mounted on a cryo-loop on a synchrotron beamline. The crystal (yellow), the cell membrane (red) and the nucleus (green) are highlighted.

Intriguingly, we noted that the inhibitory iBox appears to be duplicated in the C-terminal 22 amino acids of Inka1 (Figs 1a and 2c), which we term iBox-C. Synthetic 24mer peptides, corresponding to the amino- or C-terminal 2/3rd of the iBox or the iBox-C, exhibited Ki values of 0.2–0.4 μM (Fig. 1d), which suggested that all 38 amino acids centred on the PLV motif are involved in PAK4 inhibition. Thus, Inka1 functions as an inhibitor of kinase activity; given that it lacks sequence conservation outside these PAK4 inhibitory motifs (the iBox or iBox-C) it seems likely to be that the main function of the protein is to negatively regulate PAK4 activity. Deletion of either Inka1 or Inka2 cause subtle defects in frog and mouse development^28^,^29^, not inconsistent with human Inka1 being causative in a chromosomal microdeletion being associated with cleft lip and the central nervous system abnormalities^34^. Inka1 is expressed in a number of cell types in the early mouse embryo^28^.

### Inka1 forms crystals with PAK4 in cells

We asked whether Inka1 and PAK4 co-localize in mammalian cells (Fig. 2a). Inka1 alone is predominantly nuclear but PAK4 is not. However, co-expressing PAK4, which has been reported to contain an N-terminal nuclear localization signal^35^, redistributed Inka1 into the cytoplasm. This is interesting given the established role of PKI in terminating nuclear but not cytoplasmic PKA signals^36^. We next tested whether Inka1 inhibits active PAK4cat *in vivo*. Unexpectedly, the co-expression of these proteins consistently yielded cytoplasmic protein crystals that contained both Inka1 and PAK4, judged by immunostaining (Fig. 2b). By phase-contrast microscopy, these often appear as single elongated crystals >50 μm that extend across the cytoplasm (Fig. 2b, boxed region). Curiously, many truncated Inka1 constructs were capable of forming crystals with PAK4cat, when these contained either the central iBox or iBox-C (Fig. 2c). These crystals look remarkably similar (Supplementary Fig. 1), suggesting they have the same underlying organization. Inka1 constructs that contain both copies of the PAK4 inhibitory regions (residues 165–285) were most efficient at inducing crystals. The C-terminal 31 amino acid of Inka1 (255–285) was able to induce crystals more efficiently than the Inka1 (166–203) when they are expressed as haemagglutinin (HA)-tagged proteins, although the iBox38 has a higher affinity *in vitro*. To confirm that these crystals indeed contain a 1:1 ratio of both components, we generated a single-chain Flag–iBox–PAK4cat construct as illustrated in Fig. 2c. This expression construct yielded abundant *in-cellulo* crystals in multiple human cell types.

### The *in-cellulo* structure of Inka1 bound to PAK4cat

**Figure 3:**
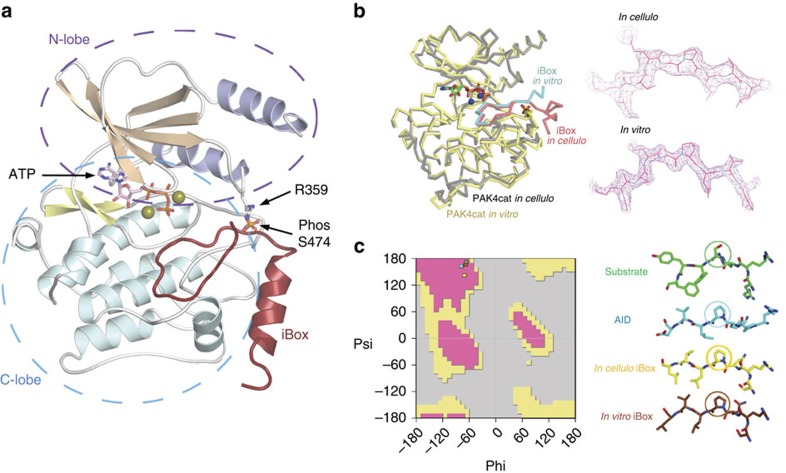
The *in-cellulo* X-ray structure of the catalytic domain of PAK4 in complex with Inka1. (**a**) The X-ray structure of the iBox–PAK4cat complex derived from diffraction of the *in-vivo* crystals. The typical kinase fold is observed with the iBox (red) binding the PAK4cat close to the phospho-Ser474 (orange), ATP and magnesium ions (mustard). (**b**) Overlay of *in vitro* and *in vivo* PAK4cat:Inka1 complex structure. Comparison between the α-carbon traces of Pak4cat:Inka crystallized *in vivo* (grey and red) and Pak4cat co-crystallized with a synthetic peptide iBox24 (see Fig. 1d). The PAK4cat with iBox24 yielded a structure at 2 Å, which was overlaid (backbone of the chains in yellow and cyan). The ATP and two Mg^2+^, found in the *in vivo* structure, are represented in stick and sphere format. On the right is the comparison of the electron density maps of the Inka1 core sequence in the two structures. Stereo images of portions of the 2Fo–Fc electron density maps contoured at 1.5*σ* and centred at P(0) in Inka is provided in Supplementary Fig. 6. (**c**) Conservation of the bond angles comparing the substrate serine with proline mimetic in Inka1. The local main-chain and side-chain orientation of the substrate serine (S0) and corresponding prolines in the substrate mimetics are as indicated. Values corresponding to these four residues mapped onto the standard Ramachandran plot indicate their similar orientation.

As the crystals of PAK4 appeared to be relatively stable within the cell, we decided not to attempt to purify these further. To tackle the *in-cellulo* crystal structure of iBox–PAK4cat, intact monkey COS-7 cells that contained large single needle crystals (<5 μm in cross-section by 50–100 μm) were trypsinized to yield rounded cells in which large crystals could be easily observed (Fig. 2d, arrows). The cells containing the largest crystals were individually mounted in cryoloops and flash frozen (Fig. 2e). These crystals were exposed to X-rays on the Diamond synchrotron microfocus beamline I24 equipped with microapertures. Typical diffraction data are given in Supplementary Fig. 2, which illustrate the importance of this micro beam to the quality of data. The merged data from five crystals led to the structure being solved at 2.95 Å resolution (Fig. 3a), the statistics for which are given in Table 1. To our knowledge, this is the first *in-cellulo* crystal structure of a mammalian protein to be elucidated within intact mammalian cells.

The X-ray structure of these *in-cellulo* crystals provided us with a number of important insights: under cellular conditions, PAK4cat adopts a typical ‘closed' active kinase conformation that includes ATP bound to two magnesium ions. As we expected, the activation (A) loop Ser474 is phosphorylated and the central region of the iBox is packed against the kinase through both main-chain and side-chain interactions (Fig. 3a). The side chain of PAK4 Arg359, which lies at the end of the αC helix, stabilizes the catalytic competent state by interacting with the phospho-Ser474. When the N-lobe αC helix is held in such a ‘closed' state with respect to the C-lobe, it allows for proper coordination of bound ATP.2Mg^2+^ for catalytic transfer. Most structures with or without substrates bound^12^ show a coupling between Arg359 and the Ser474 phosphate: the phosphorylated PAK1 Thr423 appears to use the same A-loop to phosphate coupling, to stabilize the αC helix in an active state^16^. Indeed, such coupling may well be a common mechanism feature of kinases in which activation loop phosphorylation is essential for activity, for example, PKA^37^.

On the basis of these experiments, we hypothesize that Inka1 stabilizes the ATP-bound crystallization-competent conformation of the kinase domain by preventing ATP hydrolysis through binding tightly in the cleft between the N- and C-lobes. This *in-cellulo* iBox–PAK4cat structure determined in space group P6_3_ was verified by comparison with the structure of the complex determined at 2.0 Å resolution from P4_1_2_1_2 crystals grown *in vitro* from purified PAK4cat and a synthetic iBox 24mer peptide (Fig. 3b). These two structures are essentially identical, although more of the Inka1 backbone is visible in the *in-cellulo* structure and *in vitro* structure lacks bound ATP and Mg^2+^. We are able to determine the side-chain disposition of 28 of the 38 iBox amino acids; the relative close disposition of the visible N and C termini suggest the remaining residues make intra-molecular contacts to stabilize the Inka1 inhibitor in a loop-like manner. This hypothesis is consistent with the relative Ki of the various Inka1 peptides shown in Fig. 1.

The main chain and side chains of Inka1 residues 171–196 are clearly visible with the C-terminal F191-N197 forming a helix that packs against the C-lobe (Fig. 3b). This interaction primarily involves the packing of hydrophobic side chains of Inka1, including F191, L194 and V195 against the end of the C-lobe helix α-EF and Arg488. It is likely to be that these interactions provide kinase specificity, as this region is in general more diverse. Interestingly, this part of the PAK1 C-lobe including both helix α-EF and α-G makes extensive contacts with its AID^38^, which can inhibit Pak1 with 20 nM affinity (*in trans*). Unlike Inka1, the PAK1 AID makes no contacts with the substrate-binding pocket (it is not a pseudosubstrate), but it does displace the A-loop to prevent the catalytic domain adopting an active state.

**Figure 4:**
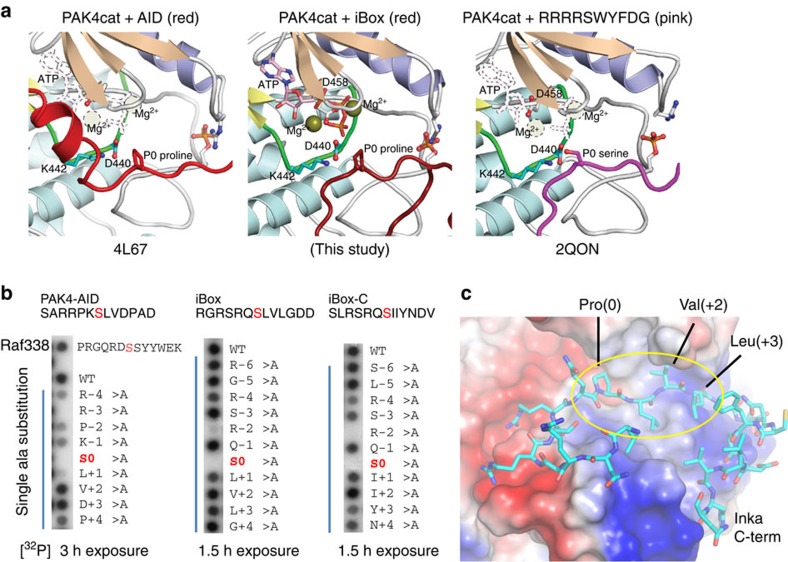
Inka1 inhibition of PAK4 activity through substrate mimicry. (**a**) Left-to-right: PAK4:AID (red); the *in-cellulo* structure of PAK4:iBox (dark red); PAK4:substrate (purple). The inhibitor prolines (P0) are similarly positioned to the serine (S0) of the substrate. (**b**) To assess the inhibitors as ‘super-substrates' we tested 13aa synthetic peptides with Pro (0)Ser substitutions in an array. The contribution of each side chain to substrate binding was assessed via alanine substitutions. The Ser (0)Ala completely abolished phosphorylation in each case, confirming other serines were not phosphorylated. (**c**) iBox-PAK4 *in-cellulo* structure highlighting the cluster of hydrophobic contacts between the Inka1 side chains and the surface of the PAK4 (yellow). The hydrogen bonds are marked in orange.

The disposition of the core Inka1 sequence (RSRQ**P**LVLGD) in the current structure shows docking in to the substrate-binding pocket (primarily via R-2 and R-4 interactions; Fig. 4c) and the inhibitor chain runs parallel to, and hydrogen bonds with, several main-chain residues of the activation loop in a β-sheet-like manner (Fig. 3a). Comparison of the PAK4-bound iBox structure (Fig. 3a,b) with that of the PAK4 AID PAK4 (ref. 33) reveals a common geometry underlying the inhibition. The iBox and AID core sequences resemble a bound consensus substrate peptide; however, the iBox and AID contain a proline residue in place of target serine designated Ser(0). Analysis of the bond angles of these residues reveals that they fall in the same region of the Ramachandran plot (Fig. 3c). It seems the relative rigidity of proline stabilizes the favourable PAK4-binding conformation of the iBox and AID peptides that mimic bound serine, thus explaining why proline was selected in both during evolution. This is different to most other intramolecular kinase pseudosubstrate sequences, for example, those in the large protein kinase C family^39^ in which the alanine is present in place of Ser(0) (RRGA(0)IKQ) in protein kinase Cα. For the well-known PKIs, an alanine occupies the Ser(0), and again basic residues at the −2 and −3 positions are critical for kinase domain interaction in the substrate-binding pocket (RRNA(0)IHD) in PKIα. The AID and Inka1 structures similarly feature Arg-mediated salt bridges that bind an acidic pocket and hydrophobic side-chain interactions at the +2 and +3 positions.

### Inka1 binds to PAK4 in a substrate-like manner

Inspection of the three structures (Fig. 4a) suggests a mechanism of phosphate transfer, similar to that proposed for the PKA^40^ and other protein kinases^41^, with PAK4 Lys442 and Asp440 from the catalytic loop, being close to the ATP γ-phosphate and Inka1 Pro(0), respectively (Supplementary Fig. 3). To test the model that these inhibitory sequences closely mimic substrate binding (Supplementary Fig. 4), we replaced Pro(0) with Ser and tested the synthetic 13mer peptides as PAK4 substrates *in situ* (Fig. 4b). The AID-based peptide was phosphorylated as efficiently as Raf1 Ser338 (ref. 13), but Inka1-derived sequences were significantly better substrates. Alanine scanning substitution showed that the presence of AID Arg(−3) or Inka1 Arg(−2) were critical for peptide phosphorylation. These side-chain contacts of Inka1 arginines (Fig. 4c) involve two acidic substrate-binding pocket (circled in Supplementary Fig. 4). Based on the phosphorylation profile both the iBox and iBox-C Arg(−4) side chains contribute significantly to peptide binding. In the PAK4:Inka1 structure the hydroxyl of the Inka1 Ser(−3) side chain forms a hydrogen bond with the Inka1 main chain; however, only in the iBox-C did we note a significant loss of interaction following Ser(−3)Ala substitution. Changing the iBox Leu(+1) and Leu (+3), which lie on a hydrophobic shoulder of the kinase, to alanine affected phosphorylation (Fig. 4b,c) as a result of reducing the side-chain hydrophobicity. Together, these observations explain the conservation of the RSRQPlvl motif among the iBox sequences (Fig. 1, upper case: invariant; lower case: positions non-bulky hydrophobic residues).

### The kinase–kinase contacts in Inka1:PAK4 crystals

**Figure 5:**
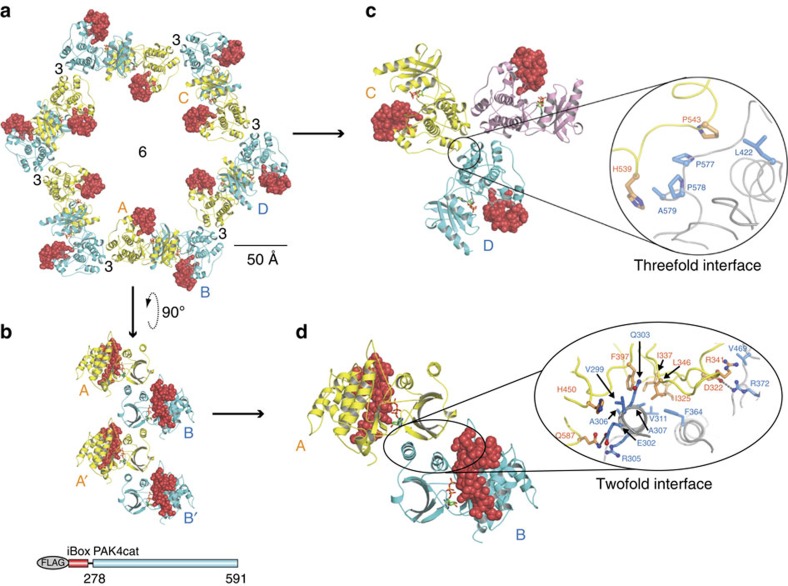
Crystal packing of the PAK4cat:Inka crystals and the nature of the protein–protein interface. (**a**) The *in-cellulo* construct and crystal packing of PAK4cat, which form the channel in the presence of Inka1 (red). The schematic of the construct is similarly coloured. (**b**) The N-lobes, which form the strands that run along the length of the channel. (**c**) The threefold axis involves hydrophobic interactions of the C-lobe, primarily involving proline residues as indicated. (**d**) The twofold interface involves primarily hydrophobic side-chain interactions between the B-subunit (blue) N-lobe α-helices including the F364 in the α-helix-C, which interacts with the β-strand sequences. The α-helix-C, a conserved feature of protein kinases, co-ordinates PAK4 kinase activity. PAK4cat (alternately yellow and cyan) and iBox (red). Numbers indicate fold axes. This schematic was generated using PyMOL Molecular Graphics System.

Inspection of the crystal packing revealed that the crystal is formed by only two types of contacts, both of which are between PAK4cat units (Fig. 5 and Supplementary Movie 1). The crystal packing resembles that obtained for a short (346 residue) isoform of full-length PAK4 (ref. 14) in which the N-terminal regulatory region is largely disordered, excepting the pseudosubstrate-like peptide (4FIG). In the *in-cellulo* crystals one set of crystal contacts is formed by the interaction between neighbouring N-lobes that involves the two helices from one N-lobe interacting with the β-sheet of the adjacent N-lobe, an interaction area of 768 Å^2^. The N-lobe interactions form strands that run the length of the crystal (Fig. 5b and Supplementary Movie 1). The hexagonal packing requires that the N-lobe be in a ‘closed' state relative to the C-lobe, which is likely to be achieved through ‘clamping' of the Inka1 inhibitory region. Interestingly, the PAK5cat sequence is slightly different at this interface and thus does not generate *in cellulo* crystals with Inka1. The second set of contacts lies at the threefold axis mediated by the PAK4cat C-lobes involving primarily hydrophobic residues; each C-lobe contributes 576 Å^2^ to this crystal contact (Fig. 5c). Remarkably, the iBox is not involved in crystal contacts and is exposed to the large 80-Å-diameter central solvent channels that run the length of the crystals (Fig. 5a). These observations thus explain the ability of multiple Inka1 deletion constructs to form crystals with PAK4, as there exists a large space to accommodate the various polypeptides associated with either iBox or iBox-C.

The packing between the N-lobes, as observed in the *in-cellulo* P6_3_ crystal form, is also reproduced in the *in-vitro* P4_1_2_1_2 crystal reported here and elsewhere^12^,^14^,^31^,^42^,^43^,^44^, and in an *in-vitro* P2_1_2_1_2_1_ crystal^45^,^46^, demonstrating that this interaction is conducive for crystallization. These two crystal forms support a range of apo peptide inhibitors and small-molecule inhibitor complexes with PAK4cat. Furthermore, both the *in-cellulo* P6_3_ threefold and N-lobe packing interactions are observed in the *in-vitro* P3 structures of PAK4 full length, PAK4cat and PAK4cat with bound peptide RPKPLVDP^14^. Thus, the two molecules in the asymmetric unit of the P3 parent crystals possess the central channel and share similar packing to the single molecule in the asymmetric unit of the *in-cellulo* P6_3_ crystals. Both P3 and P6_3_ crystals are able to accommodate larger constructs beyond the PAKcat domain that forms the entire crystal packing, namely the N terminus of PAK4 and Inka1 sequences, respectively.

### High-resolution imaging of crystal formation

**Figure 6:**
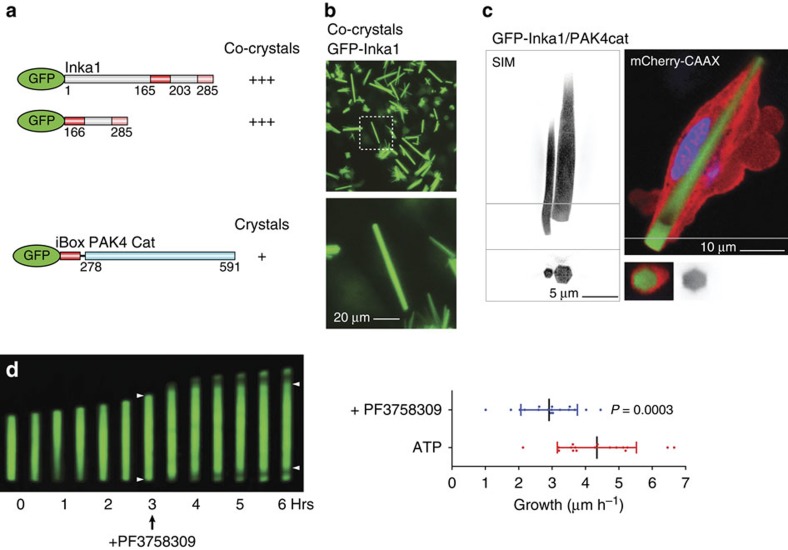
Incorporation of GFP into PAK4 crystals and their *in-vivo* dynamics. (**a**) Schematic of the fluorescent Inka1 constructs generated and (**b**) the resultant *in cellulo* crystals when transfected with PAK4cat. (**c**) Structured illumination microscopy of a cell containing two crystals (SIM, left) and a single crystal observed by two-channel confocal (right) images of GFP–Inka1:PAK4cat crystals. The cross-sections (line) show the crystal enveloped by membrane (also see Supplementary Movie 4). (**d**) Effect of addition of PF3758309 (5 μM, arrow) on a growing GFP–Inka1:Flag-PAK4cat crystal. GFP incorporation appears to occur at both ends based on the obvious depletion of GFP signal in the growing crystal after PF3758309 is added. The recovery of signal at 1.5 h after drug addition may be due to drug depletion. Right: the measured growth rates of GFP–Inka1 crystals before and after drug addition (*n*=17, error bars indicate 1 s.d.).

Based on the crystal structure described above and the available space in the lattice, we postulated that hybrid proteins of up to 30 kDa when fused to the iBox might also co-crystallize with PAK4cat *in cellulo*. Indeed, several GFP–Inka1 constructs readily formed co-crystals with PAK4cat (Fig. 6) when expressed in mammalian cells. The crystals formed with GFP–Inka1 and Flag–PAK4cat, allowed for time-lapse analysis of crystal formation (Supplementary Movies 2 and 3). By expressing the membrane marker RFP-CAAX, the plasma membrane could be observed to surround the crystal, as it exceeds the normal dimensions of the cell (Supplementary Movie 5). The co-crystallization of GFP–Inka1 and PAK4cat was modelled (Supplementary Movie 6) to demonstrate that there is sufficient scope in the PAK4cat packing to accommodate GFP. At this stage we are unable to confirm that the GFP itself is ordered sufficiently to obtain high-resolution diffraction data. Super-resolution (SIM) imaging of these GFP crystals revealed their underlying hexagonal symmetry (Fig. 6c).

As the Flag-iBox-PAK4 crystal structure contained bound ATP, which is stabilized by the Inka1 inhibitory peptide (Fig. 3a), we were interested on the effect of the ATP-competitive PAK4 inhibitor PF-03758309, which binds with 10 nM affinity *in vitro*^45^. Unexpectedly, GFP–Inka1:HA–PAK4cat co-crystals reproducibly became depleted of GFP signal during the elongation phase in 5 μM PF-03758309 (Fig. 6d). Thus, PF-03758309 appears to allow PAK4cat to incorporate with sub-stoichiometric levels of GFP–Inka1, consistent with PF-03758309 either reducing the affinity of GFP–Inka1 or allowing PAK4cat incorporation without Inka1. The average crystal growth along the length (Fig. 6d) was 4.2±1.2 μm h^−1^, which equates to adding a new layer of crystal lattice every three seconds comprising ∼50,000 protein units (for a crystal with 2 μm cross-section). Crystal growth slowed after PF3758309 addition. Based on this analysis, we observed PAK4cat incorporated at both ends of the crystal (Fig. 6d).

## Discussion

The formation of crystals or filaments in mammalian cells is unusual but not unprecedented. Depletion of ATP in cells leads to the assembly of cofilin-actin rods in various cell types including the neurons, and these rods can be purified (Minamide, 2010 #6413). The enzyme CTP synthase dynamically assembles into macromolecular filaments in bacteria, yeast, *Drosophila* and mammalian cells; it has recently been shown this might be a physiological response regulated by the non-receptor Cdc42-effector kinase DAck in the *Drosophila* embryo (Strochlic, 2014 #6412). In these two cases there is evidence that the assemblies play functional role, which has been conserved. It should be noted that PAK4 only forms crystals when it is truncated and one would anticipate such a propensity (in full-length proteins) would be selected against during evolution.

Many human protein kinases are negatively regulated via interaction of the catalytic domain with an AID^47^, but a few are also targeted by (small) inhibitory proteins, which provide an additional layer of regulation. We have identified Inka1 as a potent vertebrate inhibitor of PAK4 with a Ki of ∼30 nM (Fig. 1), which has a much higher affinity than the corresponding AID. Inka1 contains two copies of the kinase inhibitory domain and both of these small regions of themselves can support PAK4cat crystal formation in cells (Fig. 4). To our knowledge, Inka represents one of only six classes of established endogenous protein kinase inhibitors to be uncovered to date. It is likely to be that more remain to be found among the plethora of orphan open reading frames in the human genome; however, none of these different proteins share sequence homology.

**Figure 7:**
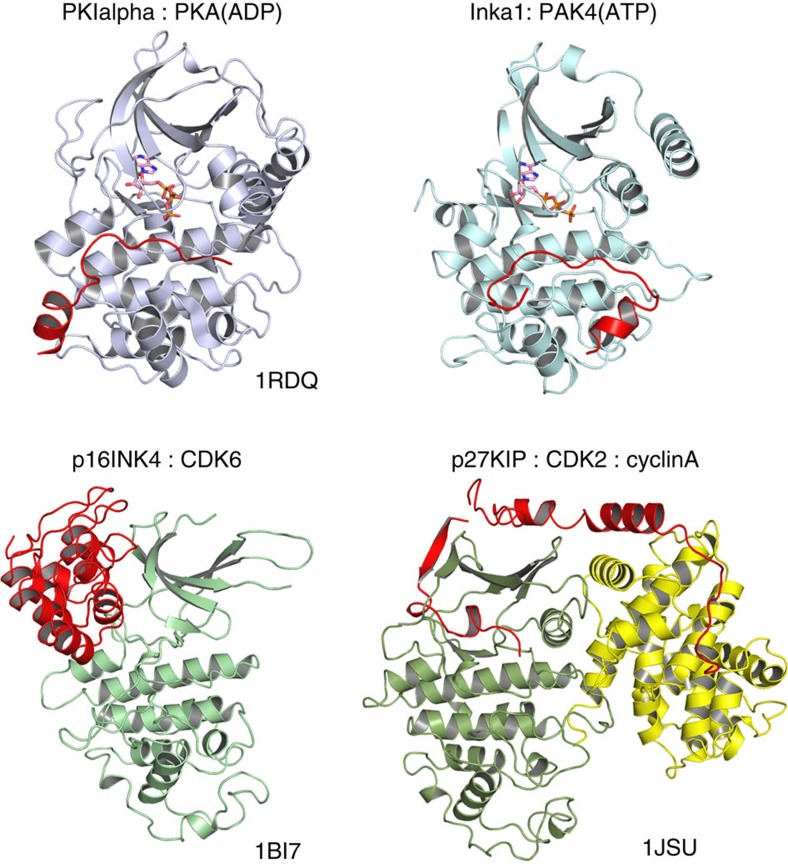
Representative structures of complexes between known classes of endogenous inhibitors and their target protein kinases. The orientation of the kinase domain (blue or green) in each case is positioned using the conserved secondary helices of the C-lobe. The organization of the inhibitor in each case is shown in red. In the case of p27 KIP, the cyclin A subunit (shown in yellow) provides an important helix to stabilize the CDK2 in an active state. It is noteworthy that the PKI and Inka1 extended region take up similar positions between the N- and C-lobes, although the helical region of each contacts are very different regions of the C-lobe.

Among known endogenous kinase inhibitors, Inka1 represents one of four whose basis of inhibition is understood at the structural level. The three members of the PKIs are proteins of <100 residues sharing an N-terminal region of 25 amino acids^48^, which interact with the PKAc catalytic domain as illustrated in Fig. 7. There is evidence that PKIγ is required for export of PKA catalytic subunits from the nucleus back to the cytoplasm following activation of PKA in the brain^36^. Based on sequence homology searches, PKI proteins can be found in many invertebrates (*cf*. K09E9.4 in *Caenorhabditis elegans*) but not in certain groups such as *Drosophila*. Two closely related Ca^2+^ calmodulin-dependent protein kinase II inhibitors (CaM-KIIN) of 78 and 79 amino acids have been characterized and show ∼50 nM Ki *in vitro*^49^.

The best-studied endogenous inhibitors are cyclin-dependent kinase (CDK) inhibitors. The *INK4* gene family encodes p16INK4a, p15INK4b, p18INK4c and p19INK4d; all bind to CDK4 and CDK6, and block their association with D-type cyclins^50^. The INK4 inhibitor structure is different from the others described here, in being well folded in the absence of kinase (Fig. 7). The Cip/Kip family members vary widely in size and comprise p21 Cip1/Waf1/Sdi1 (ref. 51), p27Kip1 (ref. 52) and p57Kip2 (ref. 53). These share a conserved N-terminal domain that binds in an extended manner to both cyclins and CDKs, as illustrated in Fig. 7. These proteins, much similar to the JIP family of mitogen-activated protein kinase scaffold proteins, are not stand-alone kinase inhibitors, but rather form a modulatory platform essential for CDK signalling^54^. Finally, the Raf1 and GRK2 inhibitor RKIP is extensively studied and its structure known^55^, but the way by which this protein binds to kinase targets is not known. Mapping studies indicate the non-catalytic domain of Raf1 binds RKIP^56^, which differentiates it from the protein kinase inhibitors shown in Fig. 7.

Both Inka1 and Inka2 are nuclear-localized proteins (Fig. 2), which can be co-immunoprecipitated with Pak4, in particular when the kinase is in an open active state. Inka proteins share sequence homology only in the region that binds to PAK4, which was termed the Inca box^17^; however, we demonstrate that Inka1 (but not Inka2) contains two related functional PAK4 inhibitory modules. There has been some discussion regarding the role of PAK4 in the nucleus, as the kinase undergoes nucleo-cytoplasmic shuttling^35^. The Inka1-LacZ allele expression in mice indicates expression in the cephalic mesenchyme, heart and paraxial mesoderm before E8.5. Subsequently, expression is observed in the migratory neural crest cells; however, the majority of Inka1−/− mice are viable and fertile^28^, pointing to compensation by Inka2. Thus, at this point we infer that Inka1 plays a redundant role in regulating PAK4 activity and may well be compensated by Inka2 in mice.

A coral fluorescent protein that forms diffraction-quality micron-sized crystals within mammalian cells has been reported in recent times^26^. These crystals assemble much more quickly and are probably recognized as foreign particles, as they are processed as autophagic cargos. By contrast, our crystals form at a modest pace in the cellular context and grow for 6–16 h (Supplementary Movies 2 and 3), suggesting they are well tolerated in the cytosol over this time period. The complex between PAK4 and Inka1 is the first human protein structure to be solved within mammalian cells and, further, multiple constructs of Inka1 or fusions to other proteins can be incorporated into the PAK4 crystal lattice (Figs 2 and 6). Crystals have been grown in a variety of mammalian cell types, monkey COS-7 and human HeLa and HEK293 cell lines (Supplementary Fig. 5).

We note parallels to the small-molecule ‘crystalline molecular flasks', which have allowed the X-ray structures of the guest molecules to be solved in host frameworks^27^. Stabilizing such guest proteins in a single state probably requires additional engineering of the channel surface, which is currently ongoing. The propensity for mammalian cells to produce single crystals using this system will allow for future structural analysis using microbeam and free-electron laser-based serial femtosecond crystallography^57^,^58^. Furthermore, the ease with which the crystals can be generated following DNA transformation into mammalian cells suggests uses in other experimental areas, such as for generating high-density *in-vivo* sensors.

## Methods

### Cloning and constructs

All plasmid constructs were generated by PCR-based DNA amplification and inserts completely sequenced. The mammalian pXJ40-based vector with Flag, HA and GFP fusion tags contain a standard cytomegalovirus-derived promoter and β–globin 5′-intron sequence^59^. Inka1 constructs were cloned in pXJ-HA (as indicated in Figs 1 and 2) or pXJ-GFP (Fig. 6), whereas PAK1 and PAK4 were cloned in pXJ-Flag. Flag–GFP–iBox–PAK4cat comprises residues 166–203 of human FAM212A (Inka1), a two-residue linker (Glu-Phe=EcoRI site) and the kinase catalytic domain of human PAK4 (278–591). For bacterial expression, pGEX4T1 (GE), pET28a (Novagen) and pSY5 (His tagged) were used as expression vectors for Inka1 (166–203), PAK1 (1–545) and PAK4 (286–591), respectively. The 13-residue peptide PAK substrate Raf1(S338) PRGQRDSSYYWEI (Raf13p) was used as previously described^13^.

### Expression and purification of recombinant proteins

Recombinant proteins were expressed in *Escherichia coli* BL21-CodonPlus(DE3) (Stratagene) grown at 30 °C. The bacteria were grown to an optical density of 0.6 (OD 600 nm) before induction with 1.0 mM isopropyl-β-D-thiogalactoside. Induction was carried out for 3 h at room temperature or 16 h at 4 °C. Bacterial lysates were purified with GSH-Sepharose (GE) or nickel Ni-NTA-Agarose (Qiagen) columns, to extract the overexpressed proteins. The recombinant proteins were eluted in 50 mM Tris–HCl, pH 8.0, 150 mM NaCl, 0.5% Triton X-100, 10% glycerol with 5 mM glutathione (for glutathione *S*-transferase (GST) fusions) or 250 mM imidazole (for poly-histidine-tagged proteins). With PAK kinases, the elution buffer was supplemented with 1 mM MgCl_2_. Proteins were diluted and snap frozen in aliquots before use. SDS–PAGE and Coomassie Brilliant Blue staining assessed protein purity to be >90%.

### Transfection of cell culture and immunoprecipitation

Monkey COS-7 cells, human HEK293 and U2OS were grown in DMEM medium with 4,500 mg l^−1^ glucose supplemented with 10% bovine calf serum (Hyclone). HeLa cells were grown in Eagle's MEM medium supplemented with L-glutamine, sodium bicarbonate, sodium pyruvate and 10% bovine calf serum. Transient transfections were performed with Lipofectamine 2000 according to recommended protocols. Typically, a total of 5 μg plasmid DNA was used per 60-mm dish; lysates were harvested 18 h later in ice-cold lysis buffer (0.5 ml; 25 mM HEPES, pH 7.3, 100 mM KCl, 5 mM MgCl_2_, 20 mM β-glycerophosphate, 5% glycerol, 0.5% Triton X-100, 5 mM dithiothreitol, 0.5 mM phenylmethyl sulfonyl fluoride, 1 mM Na_3_VO_4_ and 1 × protease inhibitor cocktail (Roche)). To test co-immunoprecipitation of proteins, the lysates were clarified by centrifugation (14,000*g*) and the clarified lysates were incubated while rolling (2 h) with 20 μl M2 anti-Flag Sepharose (Sigma-Aldrich, A2220). Rabbit anti-Flag (Sigma-Aldrich, F7425) or horseradish peroxidase-coupled anti-HA (Santa Cruz Biotechnology, sc-7392 HRP, 1 μg ml^−1^) were used for western blot analysis.

### *In-vitro* kinase assays

Purified PAK1 or PAK4 (50 nM in 25–50 μl) were incubated with 10 μM GST-Raf1S338 peptide in 10 μM ATP (2 μCi of γ32P ATP) of kinase buffer (25 mM HEPES, pH 7.3, 0.1% Triton X-100, 50 mM KCl, 10 mM MgCl_2_, 1 mM dithiothreitol) at 30 °C for 20 min. Samples were analysed by SDS–PAGE or adsorption of the GST substrate mix onto polyvinylidene difluoride membranes, followed by extensive washing to remove free γ32P-ATP. The synthetic peptides of 95% purity, as determined by HPLC and mass spectrometric analyses (GenScript), were soluble in aqueous PBS. Stock solutions (10 mM) were quantified via calculated extinction coefficients and absorbance measurements at 280 nm and stored at −80 °C. The diluted peptides were incubated at the indicated concentrations with the kinase on ice (10 min) before addition of γ32P ATP and subsequent incubation at 30 °C. The synthetic peptide array (Jerini Biotools) was phosphorylated *in situ* as described previously^60^.

### Generation and harvesting of intracellular PAK4 crystals

COS-7, HeLa, HEK293 or U2OS cells (35-mm culture dish or glass coverslip) were typically transfected with 2.5 μg of each plasmid in 2 ml of media using Lipofectamine 2000 (Invitrogen) or the GenomeONE Neo EX haemagglutinating virus of Japan envelope transfection kit (Cosmo Bio Co. Ltd) as per the manufacturer's protocol. Crystals were observed by phase-contrast microscopy using a × 10 objective (Nikon Eclipse TE300) 1–4 days post transfection. The structure of Flag–iBox–PAK4cat (Figs 2 and 3) was determined from crystals grown in COS-7 cells. The cells were harvested 3 days after transfection by incubating in PBS with 0.125% (w/v) trypsin and 25% (v/v) glycerol (Merck) for 30 min. Individual cells containing single crystals were then mounted in 0.1–0.2 mm cryoloops (Hampton Research) and flash cooled in liquid nitrogen.

### *In-cellulo* X-ray data collection and structure determination

A 2.95-Å data set was collected at the microfocus beamline I24 of the Diamond Light Source equipped with microapertures, limiting the beam cross-sectional area to 6 × 6 μm, at wavelength of 0.9686 Å with a PILATUS3 6 M detector (DECTRIS, Baden, Switzerland) by merging the diffraction data from five isomorphous crystals. The data were processed with xia2 (ref. 61) and the structure solved by molecular replacement with Phaser^62^, using the coordinates of the catalytic domain of human PAK4 (PDB 4FIE) as the search model. The solution was then built in COOT^63^, refined to completion using REFMAC5 (ref. 64) and validated via the MolProbity web server^65^. Structure figures were generated using PyMOL (The PyMOL Molecular Graphics System, Version 1.3 Schrödinger, LLC). The atomic coordinates and structure factors have been deposited in the Protein Data Bank (PDB 4XBU).

### *In-vitro* crystallization and X-ray data collection

6His–PAK4cat protein was purified under standard conditions using a semi-automated Akta system^42^. The crystallization of 6His–PAK4cat was carried by hanging drop method at 5 mg ml^−1^ with 15-fold molar excess of the iBox 23mer synthetic peptide, AEDWTAALLNRGRSRQPLVLGDW, and two times molar excess of ATP. Bipyramidal-shaped crystals grew in 0.1 M Tris–HCl, pH 8.5, 12% PEG 8000 at 25 °C. Crystals were supplemented by 15% glycerol and flash cooled in liquid nitrogen. X-ray data were collected at a wavelength of 0.9686 Å on I24 of the Diamond Light Source, and structure solution and refinement carried out as documented for the *in-cellulo* crystals.

### Live cell imaging of crystal growth and SIM and confocal microscopy

The cells were plated at 50% confluence glass cover slips overnight: plasmid transfection used GFP–iBox–Pak4cat and FLAG–iBox–Pak4cat constructs at a ratio of 4:1, to promote crystal nucleation. The cover slips were transferred to a Chamlide magnetic chamber (Live Cell Instruments, Seoul, Korea) with 5% CO_2_ at 37 °C for live imaging on an Zeiss Axiovert 200 M live-cell imaging with a × 10 objective. We imaged multiple chosen regions for 8 h at 6-min intervals. To measure crystal growth rate, we used instead a Nikon Eclipse Ti microscope equipped with spinning disk confocal attachment (Yokogawa CSU-22 module) to avoid photo-damage. The cells were imaged at × 60 1.4 numerical aperture (NA) objective at 2-min intervals. For SIM and confocal imaging, cells were fixed in non-hardening mounting media (Vectashield). The slides were imaged by Delta vision OMX SIM with a × 100 1.4 NA objective. Confocal imaging used an Olympus FV1000 upright system with a × 60 1.42NA objective. The three-dimensional stacks were analysed by IMARIS software.

## Additional information

**Accession codes:** Atomic coordinates and structure factors files have been deposited in the Protein Data Bank under accession code 4XBU.

**How to cite this article:** Baskaran, Y. *et al.* An *in cellulo*-derived structure of PAK4 in complex with its inhibitor Inka1. *Nat. Commun.* 6:8681 doi: 10.1038/ncomms9681 (2015).

## Supplementary Material

Supplementary Information Supplementary Figures 1-6

Supplementary Movie 1 Packing in iBox-PAK4cat crystals.

Supplementary Movie 2 Intracellular crystal growth. GFP-Inka1 co-expressed with FLAGPAK4cat results in the appearance of increasing numbers and size of intracellular crystals over 1-5 days and very often produced multiple crystals per cell. Crystals were observed to form over several minutes.

Supplementary Movie 3 Single crystal growth. GFP-Inka1 co-expressed with FLAG-PAK4cat vary in size, occasionally producing single large crystals per cell.

Supplementary Movie 4 3D-reconstruction. Confocal images of GFP-Inka1 and PAK4cat crystals were visualized using DiI fluorescent dye to mark the cellular membranes.

Supplementary Movie 5 3D-reconstruction. Confocal images of GFP-Inka1 and PAK4cat crystals co-expressed with mCherry-CaaX.

Supplementary Movie 6 Modeling of GFP in the PAK4cat:Inka1 lattice. The packing arrangement of iBox-PAK4cat (alternating blue and pink) molecules in the crystal with GFP (green) modeled into the large central cavity. This figure is intended to demonstrate that there is sufficient space to incorporate GFP in the channel; it is not intended to be an accurate representation of the orientation(s) of GFP in the channel.

## Figures and Tables

**Table 1:** Statistics of data collection and refinementi.

	***In-cellulo*** **PAK4cat:iBox**	***In-vitro*** **PAK4cat:iBox**
*Data collection*		
PDB code	4XBR	4XBU
Space group	P6_3_	P4_1_2_1_2
Unit cell dimensions (*a*, *b*, *c*) (Å) (*α*, *β*, *γ*) (^o^)	*a*=*b*=144.0, *c*=62.5, *α*=90, *β*=120, *γ*=90	*a*=*b*=65.2, *c*=184.2, *α*=90, *β*=90, *γ*=90
Resolution (Å)	44.2–2.94 (3.02–2.94)	29.3–2.06 (2.11–2.02)
*R*_merge_ (%)	29.4 (60.0)	7.4 (75.4)
Average I/I*σ* (%)	10.9 (2.2)	21.2 (3.9)
Unique reflections	15517	25890
Completeness (%)	97.3 (83.4)	100.0 (99.9)
Redundancy	7.8 (2.0)	12.8 (12.6)
		
*Refinement*		
Resolution (Å) (highest-resolution shell)	20.0-2.94 (3.02–2.94)	20.0–2.06 (2.11–2.06)
No. of reflections: working/test	14702/776 (906/44)	24541/1262 (1599/79)
*R*_work_/*R*_free_	18.9/23.0 (32.1/39.3)	21.1/24.7 (25.8/34.3)
No. of atoms	2536	2472
Residues PAK4/iBox	297–589/175–197	297-589/178–189
r.m.s.d. bond length (Å)	0.008	0.013
r.m.s.d. bond angle (^o^)	1.50	1.60
Mean B-factor (Å^2^) PAK4/iBox water ATP/Mg^2+^	68.9/108.9–90.2/54.0	38.6/50.3 44.0−/−
Ramachandran (%) favoured/allowed/general/disallowed	86.1/13.6/0.4/0	92.0/8.0/0/0

BOX, Inka box; r.m.s.d., root mean squared deviation
