# Supplementary material for: An in cellulo-derived structure of PAK4 in complex with its inhibitor Inka1
Source: Nat Commun. 2015 Nov 26;6:8681. doi: 10.1038/ncomms9681 (PMC4674680; doi:10.1038/ncomms9681)
Supplement: Supplementary Information — Supplementary Figures 1-6 [file ncomms9681-s1.pdf]

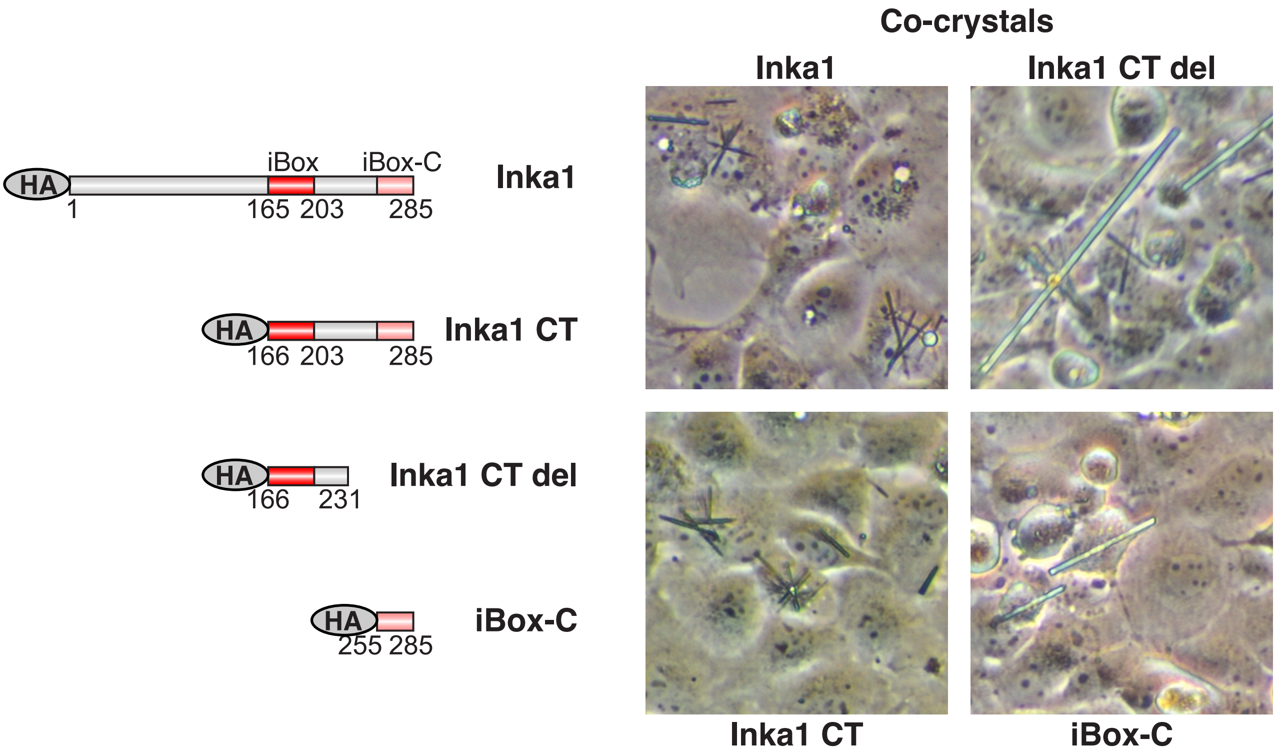

Supplementary Fig. 1 Phase contrast images of PAK4 crystals in mammalian cells. Typical fields of COS7 cells viewed by phase-contrast microscopy (x10 objective) 48h after transfection of full-length HA-Inka1 (or deletions thereof, as indicated) and co-expressed with Flag-PAK4cat.

Fig. 1

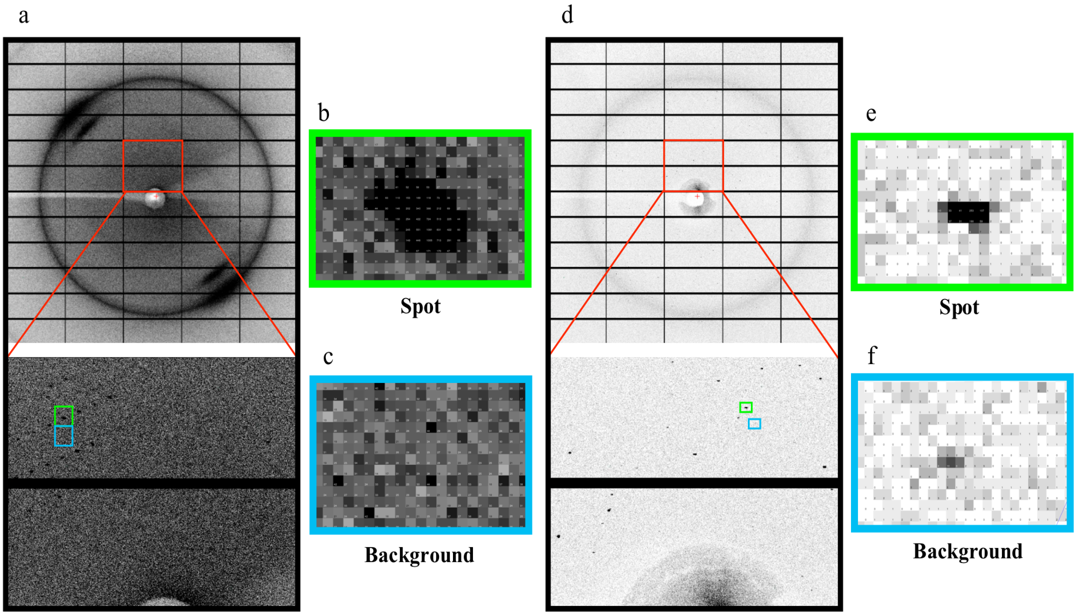

Supplementary Fig. 2. Typical diffraction data from in vivo crystals. Representative diffraction pattern of an in cellulo crystal using full beam exposure versus that with the micro-apertures. Note the relative background signal in the left image. **(a)** The full beam diffraction image with a zoomed region indicating a spot (green box) or background (blue box). **(b)** A magnified view of the spot in the green box, revealing a low signal to background signal in the image. **(c)** A magnified view of the background in the image. **(d-f)** Similar views to those presented as A-C but with micro-apertures.

Fig. 2

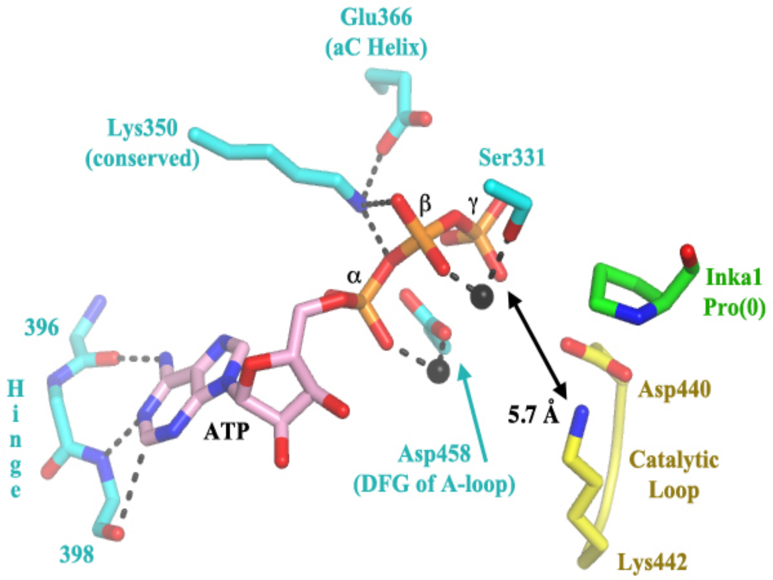

Supplementary Fig. 3. The ATP-bound active site of PAK4:Inka1. Lys442 from the catalytic loop is relatively distant (5.7 Å) to the ATP γ-phosphate in the Inka1 bound structure. PAK4 residues are shown in cyan and yellow.

Fig. 3

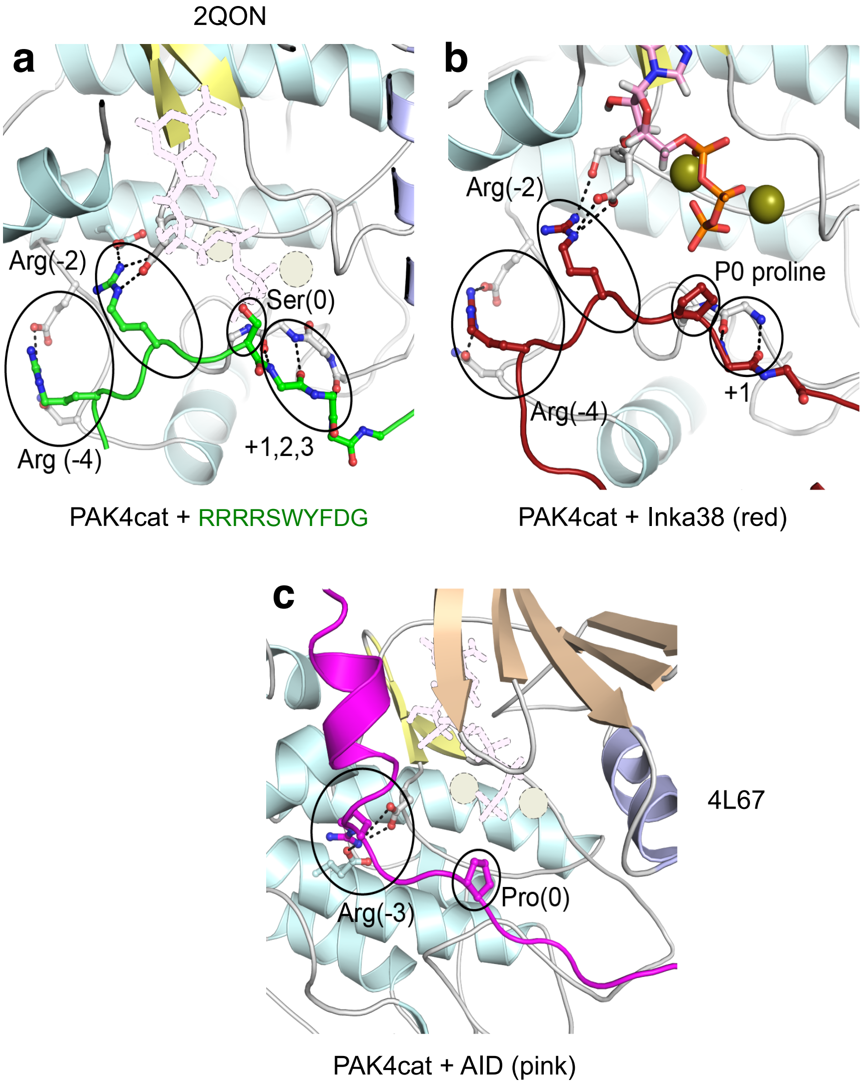

Supplementary Fig. 4. The mode of Inka1 binding to PAK4cat resembles a pseudo-substrate interaction. Structural alignment showing the key PAK4 residues involved in substrate/ inhibitor binding **(a)** A consensus substrate peptide RRRRRSWYFDG bound to PAK4cat illustrates how specific acidic pockets accommodate the side-chains of Arg (-2) and Arg (-4). **(b)** Binding interactions of iBox of the Inka1 more closely resembles substrate binding than the auto-inhibitor (AID) of PAK4 **(c)** The side-chain interaction of the AID Arg (-3) relative to proline occurs in the acidic pocket occupied by Inka1 Arg (-2) but does not contact the Arg (-4) pocket. The positions of key contacts are circled.

Fig. 4

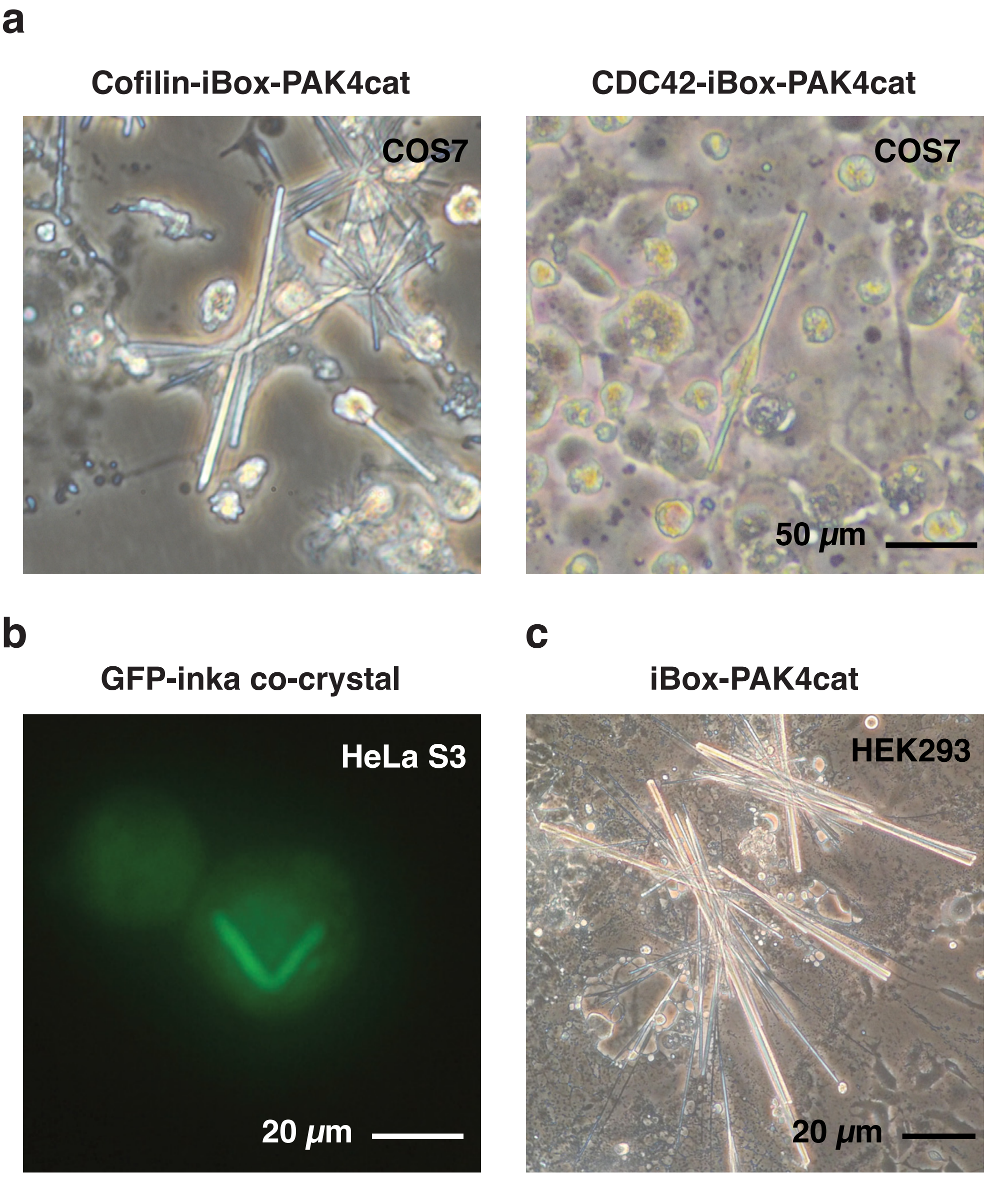

Supplementary Fig. 5 Typical *in cellulo* crystals generated in different mammalian cell types. **(a)** The micrographs show the appearance of crystals formed 48h after COS7 cells were transfected by plasmid encoding Cofilin (114D)-iBox-PAK4cat or Cdc42 (G12V)-iBox-PAK4cat fusions as indicated. **(b)** HeLaS3 were grown in suspension and transfected with plasmid encoding GFP-Inka1 and HA-PAK4cat. **(c)** HEK293 cells express and generate FLAG-iBOX-PAK4cat crystals utilizing a viral (Sendai) protein transfection system.

Fig. 5

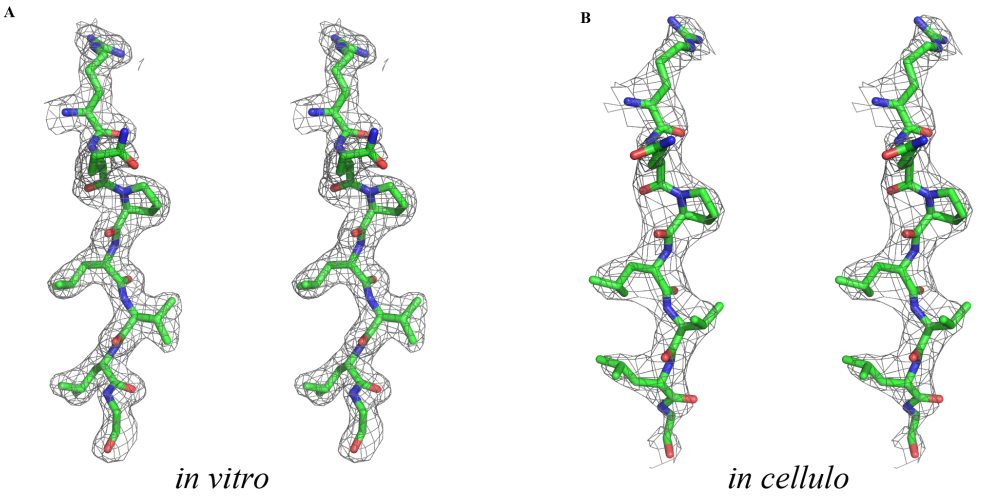

Supplementary Fig. 6. Stereo images of portions of the 2Fo-Fc electron density maps contoured at 1.5 sigma and centred at P(0) in Inka. **(a)** in vitro **(b)** in cellulo.

Fig. 6
